# Cytotoxic Properties of Some Medicinal Plant Extracts from Mazandaran, Iran

**DOI:** 10.5812/ircmj.8871

**Published:** 2013-11-05

**Authors:** Farkhondeh Nemati, Abbas Ali Dehpouri, Bahman Eslami, Vahid Mahdavi, Sepideh Mirzanejad

**Affiliations:** 1 Department of Biology, Qaemshahr Branch, Islamic Azad University, Qaemshahr, IR Iran

**Keywords:** Cytotoxins, Medicinal Plant, Iran

## Abstract

**Background:**

It was shown that plants derived agents are being used for treatment of cancer. In this study, crude ethanolic extract of *Consolida orientalis* L., *Ferula assa-foetida* L., *Coronilla varia* L., *Orobanche orientalis* G. Beck were screened in vitro for cytotoxic activity on Hela (Human cervical carcinoma) cell line.

**Objectives:**

We performed the present study to evaluate the in vitro cytotoxic activity of four plant extracts that we gathered from north of Iran, Mazandaran

**Materials and Methods:**

Hela cells were treated with various concentrations of individual samples (0.0312, 0.0625, 0.125, 0.25, 0.5, 1, 2.5, 5, 7.5 and 10 mg/ml) for 72 hours. Cell proliferation measured by MTT assay.

**Results:**

Result from the performed assay showed that ethanolic extract of *Consolida orientalis* L., *Ferula assa-foetida* L., *Coronilla varia* L. has more significant cytotoxicity effect on Hela cell line than *Orobanche orientalis* G. Beck.

**Conclusions:**

Extracts of the *Consolida orientalis* L., *Ferula assa-foetida* L., *Coronilla varia* L. could be considered as potential sources of anticancer compounds but further studies are necessary for isolation and identification of biologically active substances.

## 1. Background

Plants involve bioactive secondary metabolites and because of their complex structure, researches in this domain are notably being remarked by scientists (1, 2). Scientists collect different parts of many plants, prepare extracts, and test the extract for finding new and novel chemotherapeutics to treat cancer, as well as viral and microbial infection. Cytotoxic screening of plants is the preliminary methods to identify active compounds of plants (3, 4).

## 2. Objectives

In the course of our screening studies for the anticancer compounds from plants, we performed the present study to evaluate the in vitro cytotoxic activity of four plant extracts that we gathered from north of Iran, Mazandaran, by using human cervix carcinoma cell line, HeLa.

## 3. Materials and Methods

### 3.1. Plant Materials

Samples of *Consolida orientalis* L., *Ferula assa-foetida* L., *Coronilla varia* L., *Orobanche orientalis* G. Beck were collected from different parts of Mazandaran, Iran. Voucher specimens are deposited with the faculty of biology herbarium (as NO 720-722, 720-456, 720-036 and 720-807).

### 3.2. Preparation of Plant Extracts

The plant materials were air dried at room temperature for about 10 days and grounded into powder. Dry powder was extracted with ethanol for about 7 days at room temperature. Dry ethanolic extracts were obtained after removing the solvent by evaporation. Dry ethanolic extracts were then dissolved in dimethyl sulphoxide (DMSO) to obtain appropriate solutions of the extracts.

### 3.3. Cell Line and Culture Medium

HeLa (human cervical carcinoma) cell line obtained from Pasteur, Tehran, Iran, was used in this study. Cells were cultured in liquid medium (RPMI1640) supplemented 10% Fetal Bovine Serum (FBS), 100 u/ml penicillin and 100 µg/ml streptomycin, and maintained under an atmosphere of 5% CO_2_ and 95% air at 37^o^C (5).

### 3.4. In Vitro Assay for Cytotoxic Activity

For testing, cells were washed by phosphate buffer saline (PBS) and harvested by tripsinization and were plated in 96 well plates (one cells/well) and incubated under 5% CO_2_ and 95% air at 37^o^C for 24 hours. The cells were treated with different concentrations of plants extracts including 0.0312, 0.0625, 0.125, 0.25, 0.5, 1, 2.5, 5, 7.5 and 10 mg/ml. Dilution of stock solutions was made in culture medium yielding final extracts concentrations with a final DMSO concentration of 0.1%. This concentration of DMSO did not affect cell viability. Control cells were incubated in culture medium only. All concentrations of plants extracts were in triplicates on the same cell batch.

### 3.5. MTT Assay

Growth of tumoral cells quantitated by the ability of living cells to reduce the yellow dye 3-(4,5-dimethyl-2-thiazolyl)-2,5-diphenyl-2H-terazolium bromide (MTT) to a blue formazan product (6). At the end of 72 hours incubation, the medium in each well was replaced by MTT solution (20 cell/well, 5 mg/ml in phosphate-buffered saline), the plates were incubated for 4 hours under 5% CO_2_ and 95% air at 37ºC. MTT reagent was removed and the formazan crystals produced by viable cells were dissolved in 100 DMSO and gently shaken. The absorbance was then determined by ELISA reader at 492 nm.

The percentage growth inhibition was calculated using following formula,

% cell inhibition = 100- [(A_t_-A_b_)/(A_c_-A_b_)] × 100

Where, A_t_ = absorbance value of test compound, A_b_ = Absorbance value of blank and A_c_ = Absorbance value of control.

The effects of extracts were expressed by IC50 values (the drug concentration reducing the absorbance of treated cells by 50% with respect to untreated cells).

### 3.6. Statiscal Analysis

Experimental results are expressed as mean ± SEM. All measurements were replicated three times. The data were analyzed by an analysis of variance (P < 0.05). The IC50 values were calculated from linear regression analysis.

## 4. Results

**Figure 1.**
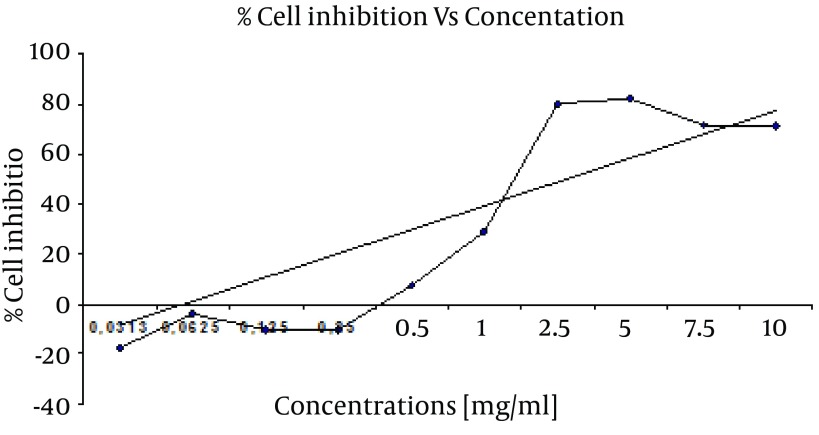
Growth Inhibition of *Consolida orientalis *L. Extracts Against HeLa Cell Line by MTT Assay

Cytotoxicity activity of four plants extracts were carried out against HeLa cell line at different concentrations to determine the IC50 (50% growth inhibition) by MTT assay. Results of different concentrations of *Consolida orientalis *L. including 0.0312, 0.0625, 0.125, 0.25, 0.5, 1, 2.5, 5, 7.5 and 10 mg/ml are tabulated in Table 1, and graphgically represented in Figure 1. MTT assay of *Consolida orientalis *L. shows significant effect on HeLa cell in concentration range between 10 mg/ml to 1 mg/ml compared with control. The highest cytotoxicity of this extract against HeLa cell was found in 5 and 2.5 mg/ml concentration with 82.45 and 80.49 percent of cell growth inhibition. It was found that the percentage of growth inhibition to be increasing with increasing concentration of test compounds, and IC50 value of this assay was 1.6 mg/ml. 

**Table 1.** Cytotoxicity Activity of *Consolida orientalis *L. Extracts Against HeLa Cell Line at Different Concentrations by MTT Assay

Concentrations of *C. orientalis* L., mg/ml	Absorbance, Mean ± SEM	Inhibition, %	IC50, mg/ml
**0.03125**	0.649 ± 0.03	-17.48	-
**0.0625**	0.578 ± 0.01	-3.94	-
**0. 125**	0.619 ± 0.04	-10.12	-
**0.25**	0.633 ± 0.09	-10.43	-
**0.5**	0.540 ± 0.06	7.59	-
**1**	0.433 ± 0.07	29.24	1.6
**2.5**	0.168 ± 0.06	80.49	-
**5**	0.155 ± 0.05	82.45	-
**7.5**	0.209 ± 0.06	71.78	-
**10**	0.215 ± 0.06	71.07	-
**Control**	0.583 ± 0.08	-	-

**Figure 2.**
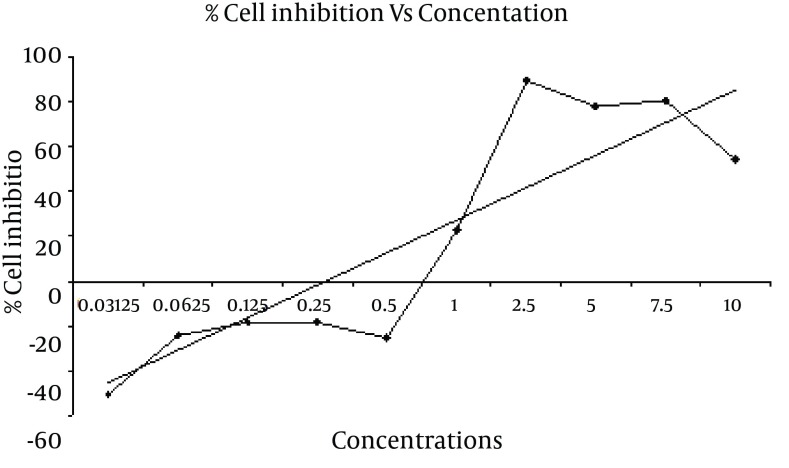
Growth Inhibition of *Ferula assa-foetida *L. Extracts Against HeLa Cell Line by MTT Assay.

Ethanolic extract of *Ferula assa-foetida *L. has significant cytotoxicity effect on Hela cell line in concentration range between 10 mg/ml to 2.5 mg/ml by using MTT assay compared with control. This extract also exerts the high cytotoxicity against HeLa cell in 2.5 mg/ml concentration with 89.65 and 80.49 percent of cell growth inhibition. IC50 value of *Ferula assa-foetida *L. on Hela cell was 0.61 mg/ml by MTT assay (Table 2, Figure 2). 

**Table 2.** Cytotoxicity Activity of *Ferula assa-foetida *L. Extracts Against HeLa Cell Line at Different Concentrations by MTT Assay

Concentrations of *F. assa-foetida* L., mg/ml	Absorbance, Mean ± SEM	Inhibition, %	IC50, mg/ml
**0.03125**	0.825 ± 0.04	-50.33	-
**0.0625**	0.706 ± 0.03	-24.33	-
**0. 125**	0.677 ± 0.02	-18.28	-
**0.25**	0.676 ± 0.09	-18.55	-
**0.5**	0.709 ± 0.008	-25.30	-
**1**	0.482 ± 0.02	22.65	0.61
**2.5**	0.155 ± 0.02	89.65	-
**5**	0.226 ± 0.03	77.76	-
**7.5**	0.213 ± 0.03	80.39	-
**10**	0.335 ± 0.03	54.19	-
**Control**	0.592 ± 0.02	-	-

**Figure 3.**
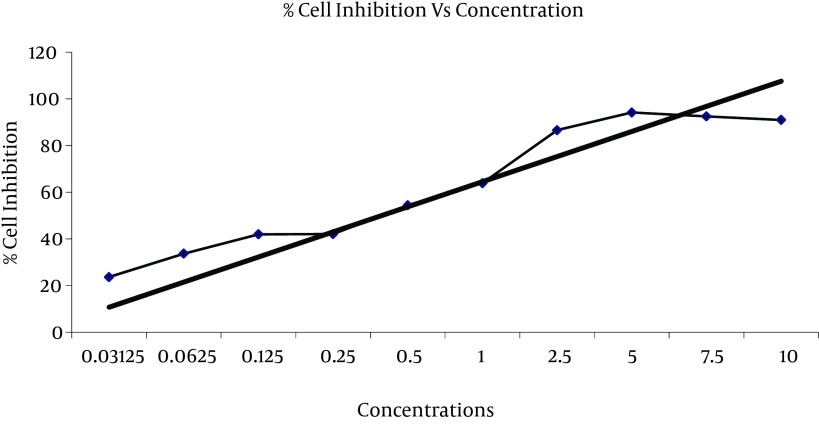
Growth Inhibition of *Coronilla varia *L. Extracts Against HeLa Cell Line by MTT Assay

Ethanolic extract of *Coronilla varia *L. has significant cytotoxicity effect on Hela cell line in concentration range between 10 mg/ml to 2.5 mg/ml by using MTT assay. The highest cytotoxicity of this extract against HeLa cell was found in 5 mg/ml concentration with 94.18% of cell growth inhibition. IC50 value of *Coronilla varia *L. on HeLa cell was 0.5 mg/ml by MTT assay (Table 3, Figure 3). 

**Table 3.** Cytotoxicity Activity of *Coronilla varia *L. Extracts Against HeLa Cell Line at Different Concentrations by MTT Assay

Concentrations of *C. varia* L., mg/ml	Absorbance, Mean ± SEM	Inhibition, %	IC50, mg/ml
**0.03125**	0.563 ± 0.01	23.63	-
**0.0625**	0.492 ± 0.007	33.70	-
**0. 125**	0.452 ± 0.02	41.95	-
**0.25**	0.451 ± 0.01	42.06	-
**0.5**	0.379 ± 0.02	54.42	-
**1**	0.326 ± 0.01	63.89	0.5
**2.5**	0.191 ± 0.04	86.59	-
**5**	0.146± 0.002	94.18	-
**7.5**	0.155 ± 0.006	92.48	-
**10**	0.161 ± 0.06	90.98	-
**Control**	0.715 ± 0.1	-	-

## 5. Discussion

**Figure 4.**
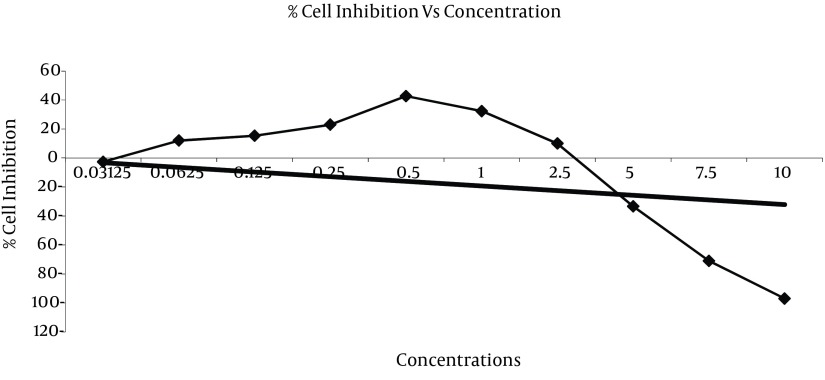
Growth Inhibition of *Orobanche orientalis *G. Beck Extracts Against HeLa Cell Line by MTT Assay

It was found that ethanolic extract of *Orobanche orientalis *G. Beck showed no significant cytotoxic ativity against HeLa cell line except in 0.5 mg/ml concentration with 42.66% of cell growth inhibition (Table 4, Figure 4). 

**Table 4.** Cytotoxicity Activity of *Orobanche orientalis *G. Beck Extracts Against HeLa Cell Line at Different Concentrations by MTT Assay

Concentrations of *O. orientalis* G. Beck, mg/ml	Absorbance, Mean ± SEM	Inhibition, %	IC50, mg/ml
**0.03125**	1.374 ± 0.14	-2.6	-
**0.0625**	1.297 ± 0.14	12	-
**0. 125**	1.263 ± 0.11	15.33	-
**0.25**	1.210 ± 0.08	23	-
**0.5**	1.093 ± 0.05	42.66	-
**1**	1.149 ± 0.05	32.33	-
**2.5**	1.271 ± 0.05	10	-
**5**	1.529 ± 0.1	-33.33	-
**7.5**	1.782 ± 0.2	-71	-
**10**	1.899 ± 0.1	-97	-
**Control**	1.341 ± 0.1	-	-

The comparison between IC50 of these extracts shows that the ethanolic extract of *Coronilla varia *L. has lower IC50 value than the others and could be considered as potential source of anticancer compounds (Table 5). 

**Table 5.** The Comparison Between IC50 of Four Extracts

IC50 ^a^, mg/ml	Hela ^a^
***Consolida orientalis***	1.6
***Ferula assa-foetida*** ** L.**	0.61
***Coronillavaria*** ** L.**	0.5
***Orobancheorientalis*** ** G. Beck**	-

^a^Drug concentration with inhibit 50% growth of cell

Overall, this study evaluate that ethanolic extract of *Consolida orientalis* L., *Ferula assa-foetida* L., *Coronilla varia* L. has potential cytotoxic activity on Hela cell, indicating the presence of cytotoxic compounds in these extracts. This study provides only basic data, further studies are necessary for isolation and identification of biologically active substances from these extracts.
